# Why does formalism exist in local environmental rectification? A qualitative comparative analysis based on Central Environmental Protection Inspection cases

**DOI:** 10.1371/journal.pone.0323134

**Published:** 2025-05-29

**Authors:** Zhou Lu, Limin Gao, Jing Wu, Xiaodong Li

**Affiliations:** School of Public Administration, Yanshan University, Qinhuangdao, Hebei, China; Bahir Dar University, ETHIOPIA

## Abstract

China’s Central Environmental Protection Inspection (CEPI) has achieved significant results, with 287,000 public reports received by June 2022, facilitating the resolution of numerous environmental issues; however, formalism persists in some regions during environmental rectification efforts. Existing research lacks sufficient explanations for the diverse driving paths underlying local formalism. This paper selects 20 typical CEPI cases from northern, central, eastern, southern, northwestern, and southwestern China. Guided by the Technology-Organization-Environment (TOE) framework, it employs the crisp-set Qualitative Comparative Analysis (csQCA) method to identify the diversified pathways of local formalism. The results indicate that: (1) Local formalism is influenced by technological, organizational, and environmental factors, including environmental facility constraints, digital facility constraints, accountability pressure, attention constraints, economic constraints, and industrial constraints; (2) No single condition constitutes a necessary cause of formalism in local environmental rectification; instead, it arises from the interaction of multiple conditions; (3) The three paths classified by nine conditional configurations, namely technology-environment path, technology-organization path, and organization path, are the primary paths for local formalism. (4) Environmental infrastructure constraints, attention constraints, accountability pressure, and industrial constraints serve as core conditions across all pathways, with accountability pressure playing a particularly significant role. This paper offers insights into mitigating formalism in local environmental rectification.

## 1 Introduction

After years of rapid economic development, China has become the world’s second-largest economy. However, environmental issues such as air pollution[1], water pollution[2], and garbage pollution[3], have grown increasingly prominent. As a unitary state, China’s environmental governance model assigns policy formulation to the central government and implementation to local governments [4]. Local governments, as policy implementers and the primary duty bearers[5], should adhere to formal institutional norms and fulfill their legal obligations in alignment with sustainable development principles. However, limited by practical factors, some local governments may deviate from institutional norms and resort to environmental governance formalism.

The formalism in environmental rectification by local governments under the CEPI has garnered scholarly attention. As an institutional innovation to enhance China’s environmental governance[6], CEPI operates under the “party-managed cadres” framework within the nation’s vertical governance structure, emphasizing “joint party-government accountability” and “dual responsibility for one position” [7]. According to official statistics, the first two rounds of the CEPI accepted 287,000 public reports, and 285,000 were resolved or were in the final implementation stage. On the one hand, CEPI has been instrumental in promoting local governments’ environmental governance. This mechanism serves as a strategic tool for policymakers to improve environmental performance[8]. On the other hand, while local governments are tasked with implementing central environmental policies, formalistic behavior has hindered their effective execution[9]. Just as environmental problems are not triggered by a single condition, the adoption of formalism is not caused by a single condition but a combination of factors. Currently, the influence of technology [10,11], organization [12], and environmental factors [13] on local formalism has been acknowledged. However, research on these factors predominantly employs a single linear approach and lacks exploration from a configurational perspective.

Building on this foundation, this study adopts a configurational perspective. Grounded in the TOE framework, using the csQCA method, it examines 20 CEPI cases and explores the multifaceted drivers underlying local formalism. The study aims to address the following issues: (1) What are the necessary conditions for triggering formalism in local governments’ environmental rectification? (2) What configurations of conditions lead to formalism in local governments’ environmental rectification? (3) What measures can effectively correct formalism in local governments’ environmental rectification practices? First, the TOE framework is applied to analyze the reasons for local formalism, laying the foundation for understanding formalism from the perspectives of technology, organization, and environment. Second, by investigating the causes from a configurational perspective, this study fills the gap in research on outcomes arising from the interplay of multiple factors in local formalism. Finally, revealing the complex driving path of formalism in Chinese regions can aid in proposing targeted measures. This can also offer insights for addressing formalism in the field of global environmental governance.

## 2 Literature review and analysis framework

### 2.1 Literature review

Formalism in local rectification is an important issue of concern in the field of global environmental governance. Currently, scholars study formalism from three primary dimensions: conceptual description, main influencing factors, and empirical evidence of negative effects.

First, conceptualization serves as the foundation for identifying formalism. Formalism is often regarded as a neutral term emphasizing adherence to form, structure, or process. However, excessive emphasis on form over practical efficacy renders formalism a pejorative term. The formalism adopted in this study carries a pejorative connotation. The pejorative conception of formalism has been conceptualized through diverse analytical lenses in scholarly discourse. From the perspective of policy implementation, Guo and Xue (2019) demonstrated that formalism is the result of policy alienation in the process of policy implementation [14]. From the perspective of central-local relations, Cui (2020) suggested that formalism is an “action-response” strategy adopted by local governments in the face of centralized campaign enforcement [15]. In terms of manifestations, some scholars argued that formalism occurs in some local governments in the form of “one-size-fits-all” and data falsification[16,17]. Zhang et al. (2020) similarly posited that formalism reflects extreme measures adopted by local governments to comply with inspections, including work stoppages, production restrictions, and shutdowns [18]. Additionally, selective governance [19], performative governance [20], and symbolic management [21], proposed by other scholars respectively, are also manifestations of formalism. Current research shows that the negative implications of formalism have received wide attention and have been defined from various perspectives. On the whole, formalism is the behavior of local governments that adopt formal means such as superficial governance, selective governance, one-size-fits-all, data falsification, etc., thereby the actual governance effect is greatly reduced.

Second, influence factors of formalism. The linear influencing factors of formalism in local rectification have been discovered by scholars. The uncertainty and complexity of environmental consolidation affect local government behavior[22]. Technology and transaction cost constraints further shape local government behavior [23]. The gaming relationship between the central government and local governments also affects local government behavior. Central accountability pressures local governments, rendering their environmental rectification efforts prone to fostering formalism. While most environmental policies are formulated by the central government, local governments retain discretion in implementing these policies[24]. Local government’s autonomy in policy implementation [25], coupled with their dual roles as economic actors and environmental regulators, incentivizes formalistic remediation based on cost-benefit analyses [26]. Collectively, scholars have analyzed formalism’s influencing factors linearly, encompassing technological, organizational, and environmental dimensions. This provides insights for this paper to deepen the research from a configurational perspective.

Third, the negative effects of formalism. Formalism is not confined to specific nations but constitutes a global challenge. This issue has undermined environmental rectification efforts. Globally, the United States exhibits conflicting federal and state pollution regulatory standards. Local governments are prone to selective enforcement amid conflicting standards [27]. Selective enforcement is a manifestation of formalism, which leads to ineffective solutions to interstate pollution problems. In Sweden, the absence of clear central guidance and local autonomy, where environmental objectives are often overridden by economic and other objectives, has made it difficult to realize the “good ecological status” of water [28]. This exemplifies the adverse consequences of formalistic selective governance.

In China, to address the policy implementation disconnect between central and local governments, CEPI has been introduced [29]. On the one hand, CEPI effectively oversees local governments’ environmental rectification efforts[30] and influences corporate behavior[31], aimed at reducing cross-border pollution[32], enhancing environmental performance, and advancing green economic development [33]. On the other hand, local governments may have “formalistic behavior”, which harms environmental rectification[34]. Kou et al. (2021) demonstrated that penalties imposed by central environmental inspections on local governments’ environmental negligence prompted across-the-board strategic rectification measures, which ultimately hindered sustainable air quality improvement [35]. Li et al. (2021) employed a breakpoint regression method to demonstrate that central supervision prompted local governments with close government-enterprise relationships to “surface-regulate” corporate emissions violations, leading to weaker regulatory outcomes compared to those with less collusion, thereby exacerbating regional disparities in environmental rectification efficiency [36]. Yang (2024) utilized an empirical approach to demonstrate that local governments, when confronted with CEPI, neither imposed stricter penalties on violators nor actively addressed pollutants excluded from routine central evaluations, thereby resulting in partial environmental rectification [37].

In summary, scholars have discussed the concept of formalism, linear influencing factors, and empirical evidence of negative effects, establishing a foundation for advancing research on local formalism. However, existing discussions on formalism’s drivers in local governments’ environmental rectification efforts rely predominantly on a single-factor linear perspective, with limited attention to the formation mechanisms arising from the configurational interplay of multiple factors. Therefore, this study adopts a configurational analysis of CEPI cases, to elucidate the multifactorial mechanisms driving formalism in local governments’ environmental rectification. By proposing corrective measures for formalism, this work aims to enrich the understanding of local deviance mechanisms under centralized governance frameworks.

### 2.2 Analysis framework

The occurrence of local formalism rectification is influenced by multiple factors such as technology, organization, and environment. Tornatzky and Fleischer proposed the TOE framework, which identifies three major factors that constrain enterprise innovation: technology, organization, and environment. The TOE framework is a systematic analytical tool. The specific forms of technology, organization, and environment vary across different industries and fields. Additionally, the framework can be used to examine how technological, organizational, and environmental factors influence behavioral patterns. Among these, technical factors refer to the adaptation relationship with the organization, such as technical facilities, technical capabilities, etc. Organizational factors are the characteristics of the organization itself, such as organizational structure and organizational culture. Environmental factors are the environment in which an organization is located, including the economic environment and legal environment. With its promotion and expansion in socio-economic research, the TOE framework is now widely applied in various fields such as government governance[38], the digital economy[39], and industrial development[40].

**Fig 1:**
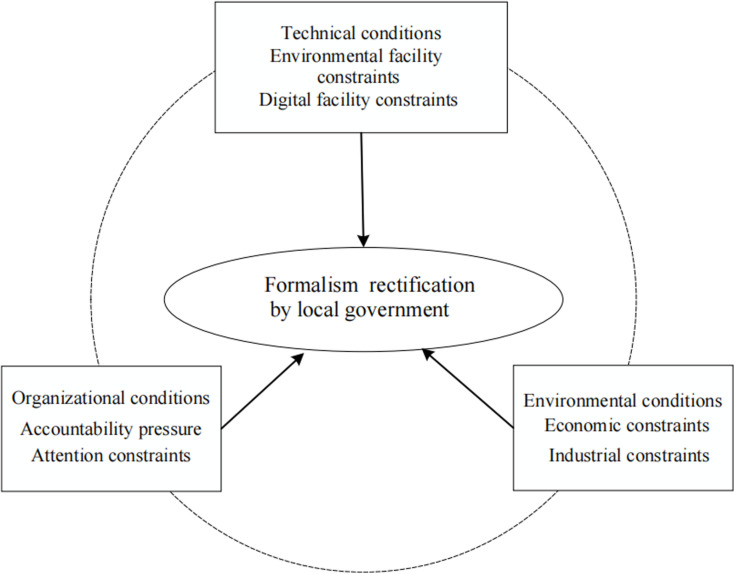
Analysis framework for formalism rectification by local government.

In the environmental field, the local formalism is the result of a combination of external socio-economic complex systems, and internal institutional and technical factors. This study attempts to introduce the csQCA method to explore the linkage effect of technical, organizational, and environmental factors on the local formalism rectification, and reveal the interactive relationship between different influencing factors. Meanwhile, based on the TOE framework, a theoretical framework was constructed by combining the institutional context of the Chinese government with environmental rectification practices, as shown in Fig 1.

First, technical conditions encompass environmental facility constraints and digital infrastructure constraints. Environmental infrastructure construction is crucial in environmental rectification. In this context, environmental infrastructure refers to context-specific facilities, such as waste disposal sites for waste pollution. In 2023, China issued the “Action Plan for Improving the Level of Environmental Infrastructure Construction (2023-2025)”[41], aiming to strengthen these systems. Environmental infrastructure is an important factor affecting the local environmental rectification. Specifically, well-developed infrastructure correlates with reduced pollution levels; even when flagged by inspection teams, local authorities can swiftly address issues or mandate corrective actions from enterprises. Conversely, inadequate or underutilized infrastructure exacerbates pollution, complicating rectification efforts post-inspection. To expedite tasks, governments may resort to informal measures for compliance. Thus, robust infrastructure accelerates rectification progress and minimizes opportunities for formalism. Otherwise, infrastructural deficiencies may compel governments to adopt formalistic rectification practices.

In digital societies, digital infrastructure construction affects the local environmental rectification behavior. Generally, with better digital facilities, a high level of big data development, and increasing symmetry of information between governments, local governments will fulfill their duties under the law due to the pressure for information transparency. Conversely, underdeveloped digital infrastructure, limited big data utilization, and asymmetric information incentivize local governments to exercise discretion in implementing environmental governance, particularly when higher-tier oversight is hindered by unreliable data. Moreover, public participation is essential for the development of a healthy environmental governance system[42], and the improvement of digital facilities provides ways for the public to participate in environmental governance. As digital infrastructure advances, the public, acting as direct observers and independent third parties, can leverage digital platforms to assess pollution levels and evaluate governmental performance objectively. This scrutiny reduces opportunities for superficial compliance by local governments. In contrast, inadequate digital tools correlate with limited public engagement, amplifying risks of formalistic rectification practices due to unchecked administrative discretion.

Second, organizational conditions encompass accountability pressure and attention constraints. Environmental accountability is an effective mechanism to urge the ecological environment to achieve orderly development through accountability[43]. Since the CEPI was implemented, supervision of organizational performance has been enforced via mechanisms like “joint Party-government accountability” and “dual-role accountability, “ resolving many challenges and advancing governance progress. However, the accountability pressure released by the “Environmental Accountability Storm” has weakened the enthusiasm and initiative of local governments, prompting them to develop a logic of self-protection and evasion of responsibility. Under this mindset, local governments tend to passively evade responsibility and adopt formalism rectification to disperse the pressure[44], which greatly reduces the effectiveness of environmental governance.

The allocation of attention reflects the concentration of the government in dealing with particular public affairs. Herbert Simon pioneered the study of organizational behavior through the lens of attention, arguing that decision-makers’ choices depend on where their attention is directed. Attention is a scarce resource, which represents the government’s attention to important matters. The scarcity of attention means that the government can only pay attention to a limited number of matters but not all matters[45]. Due to this scarcity, faced with complex public management matters, how the government’s attention is allocated determines how local governments behave. Environmental rectification, as one of the many management issues of local governments, is affected by how attention is allocated. If there is a high degree of attention in the area of environmental rectification, local governments will increase their policy implementation and actively pursue environmental rectification. Conversely, if limited attention is allocated to environmental rectification issues, local governments are likely to choose to respond to environmental rectification through formalism.

Third, environmental conditions involve economic constraints and industrial constraints.

Economic development and environmental protection are closely linked. To a certain extent, China’s environmental pollution and economic development conform to the “Environmental Kuznets Curve”. According to the “Environmental Kuznets Curve”, In the early stages of rapid economic development, a certain degree of environmental pollution is inevitable, and the limited nature of environmental resources requires increased efforts to protect the environment. When economic development reaches a critical point, it creates strong material conditions for environmental improvement. After the tipping point is passed, economic development is favorable, while environmental quality is also improved. China has been pursuing economic development for a long time, and the environment has been seriously polluted. Nowadays, under the overall layout of the country, economic development has begun to enter a stage of high-quality development. China is in a period of gear-shifting upgrading and transformation, although the ecological environment has made certain improvements, it still requires investment of time and cost in environmental rectification. The level of economic development varies from place to place, and places with a lower level of economic development are at a disadvantage when environmental rectification requires economic inputs. In addition, economic growth is the main basis for the promotion of officials. As a powerful incentive, the “promotion tournament”, which simply considers economic growth as the main path to promotion, may lead to a series of distortions in the financial expenditures or resource allocation of officials[46]. Consequently, it may lead to local governments ignoring environmental rectification and choosing formalism to maintain local economic development.

The industrial structure is closely related to green development and is an important factor influencing local environmental rectification. In particular, the secondary industry has a significant impact on environmental rectification. If the proportion of secondary industry is higher, the environmental pollution situation will be more severe, and local governments need to invest substantial time and resources in environmental protection. Conversely, if the proportion of secondary industry is relatively low, the degree of environmental pollution is relatively light, and local governments do not need to spend excessive effort on environmental rectification. China is currently undergoing industrial upgrading and transformation. The proportion of the secondary industry is tied to the success of industrial transformation. It also determines the extent of efforts local governments must invest in environmental rectification. When the proportion of the secondary industry is high, local governments face the dual pressure of industrial transformation and environmental rectification, and it is difficult for them to have the energy to carry out environmental rectification following current standards. Ultimately, local governments may resort to formalism.

## 3 Research method and data sources

### 3.1 Research method

In 1987, Ragin first proposed the Qualitative Comparative Analysis (QCA) method[47]. The QCA method, as a group comparison technique, considers cases as conditional groupings and reveals the complex causal links between conditional groupings and their outcomes, thereby answering the question, which conditional configurations are likely to lead to the desired outcome? The QCA method has been widely applied in disciplines such as sociology, political science, and public management. It examines causal relationships between social phenomena from a nonlinear perspective and is particularly suitable for small-to-medium sample analyses. The QCA method is commonly classified into three types: csQCA, mvQCA, and fsQCA. Among these, csQCA has a broad range of applications, and its selection in this study is appropriate for two reasons. First, in the context of the CEPI implementation, formalism rectification arises from a combination of technical, organizational, and environmental factors. The csQCA method can identify which combinations of factors lead to formalism rectification by local governments. Second, after the implementation of the CEPI, the official website of China’s Ministry of Ecology and Environment has published CEPI cases. The transparency and clarity of these cases provide a basis for assessing whether local governments have engaged in formalism.

### 3.2 Data collection and sources

Publicizing cases is a key step in effectively implementing the CEPI. Since its implementation in 2015, the CEPI has completed two rounds of environmental inspection work by 2022. During this period, the inspection teams publicized numerous typical cases.

This study selected 20 research samples from typical cases publicized by the CEPI team based on four principles. First, Suitability of Sample Size. The sample size should be controlled within the sample size range required by the csQCA method. The csQCA method is suitable for small-to-medium samples (10–80) and reveals multiple causal pathways. The csQCA method was chosen for this study to reveal the formation mechanism of local formalism through different combinations of conditions. Therefore, the 20 cases fulfill the csQCA’s sample size requirements. Second, Coherence in Temporal Context. The selected cases occurred during the second round of CEPI, which is relatively recent and ensures the homogeneity of the broader context at the same time. Third, Extensive Geographic Coverage with High Representativeness. Cases were selected from six major geographic regions: North, Central, East, South, Northwest, and Southwest China. The representativeness of the study was improved by further screening of typical cases in these six geographic regions. Among them, two cases in North China, Xinzhou, Shanxi Province, and Jinzhong, Shanxi Province, were selected to reflect the problem of heavy industrial pollution in the region; Six cases were selected from East China, including Huangshan in Anhui, Tongling in Anhui, Xinyu in Jiangxi, Nanchang in Jiangxi, Heze in Shandong and Jining in Shandong, covering a wide range of types of water and garbage pollution; Five cases in the Southwest region, Baoshan, Yunnan; Bijie, Guizhou; Nanchong, Sichuan; and Suining, Sichuan, were selected to exemplify the ecological protection and pollution management challenges in the region. In addition, representative cases were selected from South China, Central China, and Northwest China respectively. This geographic breadth aims to reveal regional commonalities and specificities of local formalism, providing a scientific basis for differentiated policies. Fourth, Diversity of Pollution Types. On the one hand, the csQCA approach requires the selection of a diversity of cases, which fulfills the requirements of the method. On the other hand, the main types of pollution may vary from one region to another, and the selection of cases with multiple types of pollution can improve the comprehensiveness of the study. Additionally, in examining the formation mechanisms of local formalism within the framework of the CEPI, the selection of major pollution types must necessarily align with the priority areas identified by the CEPI. Water pollution emerged as a prominent concern during the second round of inspections. Therefore, in the 20 typical cases selected, the percentage of pollution types is mainly dominated by water pollution but also includes garbage pollution, mine pollution, soil pollution, and air pollution.

The case selection process followed three steps: First, cases published during the second round of the CEPI were collected through official channels (e.g., the CEPI website, local government portals, and news reports), forming a preliminary case pool. Second, 20 representative cases were selected based on the four principles outlined above. Finally, relevant data released by local governments during the second inspection cycle were compiled to ensure data comprehensiveness, reliability, and usability.

Based on the above principles and steps, 20 typical cases that occurred during the second round of CEPI in 2021 were selected, as shown in Table 1.

**Table 1:** Case location and environmental pollution types.

Districts	Types	Districts	Types
Baoshan City	Water pollution	Xianyang City	Air pollution
Tieling City	Water pollution	Nanchong City	Water pollution
Xinyu City	Mine pollution	Xinzhou City	Garbage pollution
Huangshan City	Water pollution	Chongzuo City	Water pollution
Zhuzhou City	Water pollution	Jinzhong City	Water pollution
Xinxiang City	Garbage pollution	Xiaogan City	Water pollution
Maoming City	Water pollution	Suining City	Soil pollution
Heze City	Water pollution	Zhongshan City	Water pollution
Bijie City	Water pollution	Nanchang City	Water pollution
Tongling City	Water pollution	Jining City	Mine pollution

## 4 Results and discussion

### 4.1 Variables setting and truth table construction

The setting and assignment of the result variable and the condition variables are the prerequisites for the analysis using the csQCA method. The csQCA is the method of binary assignment. If it is within the range, it will be assigned as 1, otherwise it will be set as 0. Moreover, in most cases, the average value or median value will also be considered as the reference standard for the csQCA assignment. Based on this rule, after analyzing the corresponding data, the result variable and the condition variables can be assigned binary values respectively. The relevant data come from information published on various official websites in China, including typical cases of CEPI published by the Ministry of Ecology and Environment, government work reports, and the Statistical Bulletin of National Economic and Social Development published by various local governments, as shown in Table 2.

**Table 2:** Setting of Variables.

Condition Variables	Variables discrimination	Assignment	Data sources
Environmental facility constraints	Environmental facility issues exist	1	Official case report
	No environmental facility issues	0	
Digital facility constraints	The number of Internet broadband connections per capita in 2020 is below average	1	Urban Statistical Yearbook
	The number of Internet broadband connections per capita in 2020 is higher than average	0	
Accountability pressure	Higher than average accountability per capita	1	Official Case Report Urban Statistical Yearbook
	Lower than average accountability per capita	0	
Attention constraints	Below-average keyword “environment” in the 2020 government work report	1	Government Working Report
	Above-average keyword “environment” in the 2020 government work report	0	
Economic constraints	The GDP growth rate in 2020 is lower than the average	1	Statistical Bulletin on National Economic and Social Development
	The GDP growth rate in 2020 is higher than the average	0	
Industrial constraints	The proportion of the secondary industry in 2020 exceeded 40%	1	Statistical Bulletin on National Economic and Social Development
	The proportion of the secondary industry in 2020 is less than 40%	0	
Result Variable	Formalism rectification exists	1	Official case report
	No formalism rectification	0	

#### 4.1.1 Setting of the result variable.

The formalism rectification of local governments in environmental inspection is used as the result variable. The second round of the CEPI revealed a large number of typical notification cases, including some typical cases of perfunctory rectification, data falsification, and other formalist behaviors. At the same time, some typical cases, although notified, are based on the lack of cognitive ability and other reasons, rather than based on formalism rectification behavior. Based on comprehensive evaluation and analysis of typical cases, the value assigned to those who take formalistic environmental rectification is 1, and the value assigned to those who do not take formalistic environmental rectification is 0.

#### 4.1.2 Setting of condition variables.

Environmental facility constraints: Well-established environmental facilities can reduce the difficulty of environmental rectification and reduce the scope for local governments to “maneuver” or “operate informally”. If there are difficulties in the normal use of environmental facilities, local governments can easily fall into the space of “flexibility” or “informal operation” under the pressure of inspection and accountability. Different ecological environments require different infrastructure, such as the differences in environmental infrastructure for dealing with water and air pollution. The assignment of values for environmental facility constraints is mainly based on the notification of typical cases of the CEPI. If there are cases of incomplete or improperly applied environmental infrastructure in the notification of the CEPI, the value is assigned to 1, otherwise, the value is assigned to 0.

Digital facility constraints: The construction of robust digital facilities can address the issue of information asymmetry between higher and lower levels of government, enabling the public to access information on local environmental rectification and thereby regulating government behavior. Poorly constructed digital facilities leave little room for upward and downward oversight, as well as public scrutiny, and local governments are likely prone to formalism rectification. The number of broadband internet access per capita can reflect the construction of digital infrastructure in a city. Concerning the studies of some scholars[48], the average number of internet broadband households per capita in the city is used to represent the level of digital facilities in the city. Higher than average, with higher levels of digital facilities; Below average, the level of digital facilities is relatively low. If the per capita number of internet broadband access households in the city where the case is located is lower than the average value, the assigned value is 1, otherwise, the assigned value is 0.

Accountability pressure: Accountability pressure affects local environmental rectification behavior. The higher the accountability pressure, the higher the probability that local governments will choose formalism. After cases are reported by the CEPI team, local governments subsequently announce the number of people held accountable in a given case. Referring to the previous research method of taking the degree of accountability and punishment as an alternative variable of accountability pressure[49], this paper uses the per capita number of accountability cases as a proxy indicator of environmental accountability pressure. Higher than the average value of accountability per capita means greater pressure on accountability. The allocation value is 1, otherwise it is 0.

Attention constraints: Local governments are in charge of many public affairs, and the scarcity of attention determines that local governments can only focus on special matters. Since “attention” itself, as an abstract concept, needs to be presented in a certain vehicle, concrete indicators should be chosen for its measurement. The specific way is to examine the government’s attention by mining and analyzing the content of local government work reports[50]. The local government work report is the annual summary and outlook of government work, which undoubtedly reflects the government’s psychological expression and attention allocation. Taking the 2020 Government Work Report of the city where the case is located as the text basis, the frequency of “environmental” keywords is calculated using the software as an alternative indicator for attention allocation. The number of keyword word frequencies below the average is assigned a value of 1 and vice versa is assigned a value of 0.

Economic constraints: The balance between economic development and environmental protection has always been an important topic of concern for local governments. After a long period of development in which the economy was emphasized over environmental protection, China has begun to move into a stage of high-quality development in which economic development and environmental protection are given equal importance. Since the traditional development concepts of some local governments have not kept pace with the times, coupled with the low level of local economic development that cannot support the efforts to rectify the environment, the local governments will likely adopt formalism to carry out environmental rectification. The annual GDP growth rate reflects a region’s level of economic development for the year. As a result, the 2020 GDP growth rate of the case location below the average is assigned a value of 1, and vice versa is assigned a value of 0.

Industrial constraints: In the context of deepening industrial reform, the higher the proportion of the secondary industry, the more difficult it is to upgrade and transform the industry. The greater the difficulty of industrial upgrading and transformation, the more constrained the environmental rectification of local governments, and formalism has become a “rational choice” for local governments. Therefore, the proportion of secondary industry is used to measure the degree of industrial constraints. Meanwhile, based on the collection and organization of the proportion of the secondary industry in case cities in 2020, 40% is the boundary. If the proportion of the secondary industry in a city is higher than 40%, it is assigned a value of 1, otherwise, the assigned value is 0.

#### 4.1.3 Truth table construction.

After collecting the data and assigning values to the result variables and condition variables, the results of all variables are obtained to form the truth table, as shown in Table 3.

**Table 3:** Truth Table.

Districts	Environmental facility constraints	Digital facility constraints	Accountability pressure	Attention constraints	Economic constraints	Industrial constraints	Formalism rectification
Baoshan	1	1	1	0	0	0	1
Tieling	1	1	1	1	1	0	1
Xinyu	1	0	1	1	0	1	1
Huangshan	0	0	1	0	1	0	1
Zhuzhou	1	1	0	0	0	1	1
Xinxiang	1	0	1	0	0	1	1
Maoming	1	1	0	0	1	0	0
Heze	1	1	0	1	0	1	0
Bijie	1	1	0	1	0	0	1
Tongling	0	0	1	1	0	1	1
Xianyang	1	0	1	1	1	1	0
Nanchong	0	1	0	0	0	0	0
Xinzhou	0	1	1	0	0	1	1
Chongzuo	1	0	0	1	0	0	1
Jinzhong	1	0	1	1	1	1	0
Xiaogan	1	1	0	0	1	0	0
Suining	0	1	1	1	0	1	1
Zhongshan	1	0	0	1	1	1	0
Nanchang	1	0	1	0	0	1	1
Jining	0	1	0	1	0	0	1

### 4.2 Univariate necessity analysis

In constructing the truth table, it is essential to verify the absence of conflicting configurations to ensure their exclusion from the table. After verifying that there are no conflicts in the data in the truth table, two important aspects of the csQCA methodology begin, namely univariate necessity analysis and conditional configuration analysis. Univariate necessity analysis is the process of determining whether there are necessary conditions that lead to the occurrence of the result variable. A necessary condition is a type of condition that must exist after the result occurs. In the QCA method, the necessary conditions are usually judged by consistency and coverage. The judgment criterion is that when the consistency of a condition is higher than 0.9, it is considered to be a necessary condition that leads to the occurrence of the result variable[51]. Coverage refers to the explanatory validity of the condition variable, the higher the coverage, the stronger the explanatory power of the variable.

Use fsqca4.1 software to analyze the univariate necessity of the truth table, and the results of univariate consistency and coverage are shown in Table 4. As can be seen from Table 4, there are a total of 12 variables in the two cases where the superposition of 6 conditional variables exists or does not exist, the consistency of each conditional variable is less than 0.9, and there is no case higher than 0.9. It means that technological, organizational, and environmental conditions such as environmental facility construction, digital facility construction, accountability pressure, attention allocation, economic development level, and even industrial structure are not necessary conditions for local governments to formalism rectification. In other words, no single factor can promote the local government to produce formalist rectification in the CEPI. The factors that influence local governments to choose formalism in environmental rectification are multiple and are the result of a combination of technological, organizational, and environmental factors.

**Table 4:** Univariate necessity analysis.

Conditional variables	Consistency	Coverage	Conditional variables	Consistency	Coverage
Environmental facility constraints	0.62	0.57	Attention constraints	0.54	0.64
~Environmental facility constraints	0.38	0.83	~ Attention constraints	0.46	0.67
Digital facility constraints	0.54	0.64	Economic constraints	0.15	0.29
~ Digital facility constraints	0.46	0.67	~ Economic constraints	0.85	0.85
Accountability pressure	0.69	0.82	Industrial constraints	0.54	0.64
~ Accountability pressure	0.31	0.44	~ Industrial constraints	0.46	0.67

### 4.3 Conditional configuration analysis

From the univariate necessity analysis, it can be found that formalism is not the result of a single condition, but rather the composite effect of multiple internal and external conditions, including technology, organization, and environment. Unlike univariate necessity analysis, which explores whether each variable is a necessary condition for the result variable, configuration analysis focuses on the adequacy of the combination of multiple conditions leading to the occurrence of the result variable. Using the fsQCA4.1 software, the consistency threshold is set to 0.8, the frequency threshold is set to 1, and the standard analysis is conducted on the truth table. Through the standard analysis, three kinds of operation results are obtained: simple solution, intermediate solution, and complex solution. Intermediate solutions allow for the inclusion of logical residuals and better reflect the many forms that combinations of conditions can take, choosing intermediate solutions for analysis is appropriate. Based on the intermediate solution, a single condition or combination of conditions appearing in both the intermediate solution and the simple solution is identified as the core condition.

The intermediate solution obtained through analysis includes nine conditional configurations, each of which causes the occurrence of formalism in a “different path and the same destination” manner, as shown in Table 5. In Table 5, configurations with different core conditions, such as Configuration 3a and Configuration 4c, result in the occurrence of result variables in a “different path and the same outcome” manner. Configuration 3a takes “attention constraints” and “non-industrial constraints” as the core conditions. At the same time, “environmental facility constraints” and “non-economic constraints” are the edge conditions. In Configuration 3a, the local economy develops rapidly and the industrial structure is reasonable, but the environmental attention invested by local governments is insufficient, coupled with the influence of the construction of environmental facilities, local governments engage in formalism rectification. The formalism rectification of Bijie City and Chongzuo City in the CEPI is mainly based on this driving path. Configuration 4c takes “accountability pressure” and “non-economic constraints” as the core conditions, and takes “non-environmental facility constraints”, “digital facility constraints” and “industrial constraints” as the edge conditions. This configuration, with good local environmental infrastructure and better economic development, but with a crude industrial structure and a low level of digital development, has given rise to local governments choosing formalism rectification when accountability pressure plays a core role. The formalism rectification in Xinzhou and Suining is mainly based on this driving path. Configurations 3a and 4c can prove that each conditional configuration leads to formalism rectification behavior differently. In addition to different core conditions causing the same outcome to occur, groupings with the same or similar core conditions also cause the result variable to occur in the same way, such as Configuration 3a, Configuration 3b, and Configuration 4a, Configuration 4b.

**Table 5:** Conditional configuration analysis.

	Technology-environment type	Technology-organization type	Organization type						
			Attention constraints		Blame-avoidance thinking drive				
**Condition variables**	1a	2a	3a	3b	4a	4b	4c	4d	4e
**Environmental facility constraints**	●	●	^●^		⊗	^●^	⊗	^●^	
Digital facility constraints	^●^	^●^		^●^	⊗	⊗	^●^	^●^	⊗
Accountability pressure		●			●	●	●	●	●
Attention constraints	**⊗**	**⊗**	●	●				^●^	^●^
Economic constraints	**⊗**	**⊗**	⊗	⊗	^●^	**⊗**	**⊗**		**⊗**
Industrial constraints	●		**⊗**	**⊗**	**⊗**	^●^	^●^	**⊗**	^●^
Raw coverage	0.08	0.08	0.15	0.15	0.08	0.23	0.15	0.08	0.15
Unique coverage	0.08	0.08	0.08	0.08	0.08	0.15	0.15	0.08	0.08
Consistency	1	1	1	1	1	1	1	1	1
Solution coverage	1								
Solution consistency	1								

*Notes:*●or^●^ represents the existence of a condition;**⊗**or⊗ represents the absence of a condition;●or**⊗** represents the core condition;^●^or⊗ represents the edge condition, and the blank table represents the conditions that have no impact.

Considering the existence of the same or similar core variables in some conditional configurations, the nine conditional configurations were categorized using this criterion, which resulted in three types of role pathways: technology-environment type, technology-organization type, and organization type. Among the nine conditional configurations, the consistency of the solutions is 1, and the coverage of the solutions is 1, indicating that the conditional configuration results are more explanatory and cover 100% of the cases.

Technology-Environment Type: The technology-environment model is 1a, with “environmental facility constraints”, “industrial constraints”, “non-attention constraints” and “non-economic constraints” as the core conditions, and “digital facility constraints” as the edge conditions. In configuration 1a, the consistency is 1, and both the raw coverage and unique coverage are 0.08, indicating that configuration 1a has strong explanatory power, covering 8% of cases, and 8% of cases can only be explained by this path. Under the technology-environment model, the local economy develops well, and the local government also pays attention to ecological environment protection. However, due to the severe constraints of environmental infrastructure and secondary industry, local governments are inclined to opt for formalistic rectification. Additionally, the lack of digital facilities and the obstruction of external monitoring channels have, to some extent, influenced the behavioral choices of local governments.

The city of Zhuzhou in Hunan Province adopts formalism under the technology-environment model. Zhuzhou is located in the north-eastern part of Hunan Province, downstream of the Xiang River. Its unique geographical location requires Zhuzhou City to strengthen environmental infrastructure construction, but the lag in sewage pipe network renovation and water pollution control poses significant challenges. Zhuzhou City is one of the first eight key industrial cities built after the founding of New China, is China’s old industrial base, in the rapid economic growth while the industrial structure is seriously unreasonable, the proportion of the secondary industry in 2020 to reach 46.3%. The environmental infrastructure is inadequate, the proportion of the secondary industry is too high, and local governments face difficulties in environmental rectification. Additionally, given the lag in digital facilities and limited external monitoring pressure, relevant departments in Zhuzhou City have opted for formalistic responses to the CEPI.

Technology-Organization Type: The corresponding configuration of the technology-organization type is 2a, which takes “environmental facility constraints”, “accountability pressure”, “non-attention constraints” and “non-economic constraints” as the core conditions, and “digital facility constraint” as the edge conditions. The consistency of Configuration 2a is 1, indicating strong explanatory power. The raw coverage and unique coverage are 0.8, indicating that 8% of the cases are covered, and 8% of the cases can only be explained by Configuration 2a. Under the technology-organization model, environmental facility constraints and accountability pressure play a crucial role in the process of motivating local governments to choose formalism, while digital facility constraints serve a supplementary role.

The city of Baoshan in Yunnan Province exhibits formalism in response to the CEPI, primarily following this action path. The sewage treatment capacity in the main urban area of Baoshan City is seriously inadequate, and the environmental infrastructure is notably deficient. Approximately 45,000 tons of sewage are discharged directly into the East River daily, causing it to become heavily polluted. According to official data, the per capita number of individuals held accountable for environmental rectification in Baoshan City exceeds the average, reflecting a high level of environmental accountability. The lack of environmental infrastructure, and excessive accountability, coupled with the lack of digital facilities and weak multiple supervision, constitute the main driving path for relevant departments to generate formalism in Baoshan City.

Organization Type: Local environmental rectification behavior is influenced by organizational factors. When constrained by these factors, local governments are prone to formalism. As shown in Table 5, the organization type can be categorized into two specific modes: attention constraints and blame-avoidance thinking drive.

Attention Constraints Model: This model includes configurations 3a and 3b. The consistency of the two conditional configurations is 1, and the raw coverage is 15% respectively, which means that the attention constraints model has strong explanatory power and covers 30% of cases. Configurations 3a and 3b share “attention constraints” and “non-industrial constraints” as core conditions. Additionally, configuration 3a includes “environmental facility constraints” and “non-economic constraints” as edge conditions, while configuration 3b includes “digital facility constraints” and “non-economic constraints” as edge conditions. Under the attention constraints model, the local industrial structure is reasonable, but the local government does not pay enough attention to environmental protection, which is manifested as insufficient or unfulfilled attention to environmental protection. The scarcity of attention determines that local governments cannot devote all their attention to environmental rectification, and when attention is limited or not put into practice, it is easy for local governments to form formalism to pursue other tasks such as economic development. Furthermore, the constraints of environmental infrastructure and digital infrastructure will to some extent increase the probability of local governments choosing formalism.

The formalism in environmental rectification in Jining City, Shandong Province, primarily follows this action path. Jining is located in the “Three-year Action Plan to win the BlueSky Defense War” clear key areas, but also in Shandong Province, the seven Beijing-Tianjin-Hebei and surrounding areas of air pollution transmission channel cities. According to the 2020 Government Work Report of Jining City, the frequency of keywords related to “environment” is lower than the average, indicating that the government’s investment in environmental attention is relatively low and the degree of emphasis on environmental governance is insufficient. Faced with the CEPI, the scarce attention of relevant departments in Jining City has not been focused on environmental rectification, but on economic development or other aspects, which is an important incentive for its formalism to occur.

Blame-Avoidance Thinking Drive Model: The motives of government officials can be categorized into three types: claiming credit, good governance, and blame avoidance. Their behaviors are not characterized by the pursuit of maximizing merit in the traditional sense, but by seeking to minimize responsibility as much as possible[52]. Due to this shift in motivation, local governments are prone to blame-avoidance thinking when accountability pressure becomes excessive. This represents a self-protective coping logic adopted by local officials to evade action, blame, or change[53]. Blame-avoidance thinking exists in political and organizational life[54]. Under the operation of the CEPI, some local governments take perfunctory rectification, surface rectification, and other formalism coping methods, which are essentially the result of avoiding responsibility thinking.

According to Table 5, the blame-avoidance thinking drive model includes Configurations 4a to 4e. These five configurations with a consistency of 1, and a total raw coverage of 0.69, which means that the blame-avoidance thinking drive model is highly explanatory and covers 69% of the cases. The raw coverage of the five configurations is not the same, the raw coverage of Configurations 4b, 4c, and 4e are 0.23, 0.15, and 0.15 respectively, covering 53% of the cases, which is representative. Among the five conditional configurations, accountability pressure is a common core condition, playing a central role in local government environmental rectification and serving as a key driver for formalism. Other core conditions also play a key role in the five configurations, such as the core condition “non-industrial constraints” in Configurations 4a and 4d, and the core condition “non-economic constraints “in Configurations 4b, 4c, and 4e. In addition to the core conditions, the five configurations have different edge conditions that play a supplementary role. In the case of Configuration 4b, “environmental facility constraints”, “non-digital facility constraints” and “industrial constraints” are edge conditions, which means that the local formalism is not only constrained by accountability pressure but also to a certain extent by the configuration of the local environmental infrastructure and industrial structure.

Xinxiang City, Henan Province, has exhibited formalism during the CEPI, primarily driven by the blame-avoidance thinking drive model. Xinxiang City is a major industrial town in northern Henan, Xinxiang’s industrial structure is crude, and although the speed of economic development is relatively fast, the environmental infrastructure is insufficient, so the local government in the rubbish pollution control is stretched to the limit. Moreover, the per capita accountability rate for inadequate waste management in Xinxiang City is higher than the average, which means that the relevant departments in Xinxiang City bear a greater level of accountability in the second round of environmental inspection. This strong level of environmental accountability serves as the core driver for the local government’s formalism.

### 4.4 Robustness test

The method of adjusting the consistency threshold is relatively universal, making it suitable for robustness testing. Using the fsQCA4.1 software, the initial consistency threshold of 0.8 was increased to 0.85. After this adjustment, the resulting configurations remained consistent with the previous findings, confirming the robustness of the test results.

### 4.5 Discussion

According to the study’s findings, some local governments have exhibited formalistic rectification during the CEPI. The causes of which are not caused by a single condition, but by a composite of technical, organizational, and environmental conditions. The technology-environment path, technology-organization path, and organization path, which are constituted by the conditions of technology, organization, and environment, are the main action paths for local formalism rectification. Environmental infrastructure constraints, industrial constraints, attention constraints, and accountability pressures are the core conditions distilled from the three driving paths, with accountability pressure playing a particularly significant role.

Existing studies provide supporting evidence for the findings of this paper. Wang et al. (2021) highlighted that in the process of the CEPI, local governments do have problems with non-correction and negative correction [55]. In other words, the use of “response” behavior to cover up the facts [56], which is essentially the local government did not perform their duties by the law [57]. By analyzing the case of the CEPI, Xue (2024) noted that inadequate environmental infrastructure can adversely hinder precision policymaking [58], which is consistent with the conclusion of this paper that environmental infrastructure limitations serve as a core condition. Zheng et al. (2019) emphasized the close relationship between industrial structure and environmental governance effectiveness, supporting this paper’s findings on industrial structure constraints [59]. Wang & Ma (2024) found that the stronger the attention, the better the environmental governance effect. The weaker the attention, the worse the environmental governance effect, which verifies the importance of attention allocation on the environmental governance effect and echoes the attention condition proposed in this paper [60]. In addition, in terms of accountability pressure, Zhu et al. (2024) demonstrated the impact of responsibility-avoidance thinking formed under the accountability pressure of CEPI on local formalism [61]. Guo (2023) further emphasized the importance of precise accountability to avoid the problem of formalism [62]. This coincides with the finding in this paper that accountability pressure is the core driver of local formalism and occupies a large proportion.

## 5 Conclusion and prospects

### 5.1 Conclusion

Under the operation of CEPI, local governments are expected to actively engage in environmental rectification. However, in practice, some local governments fail to do so. Why does formalism occur in local environmental rectification? This is a critical issue worth exploring. To address this, focusing on the theme of why formalism arises in local environmental rectification, this paper employs the TOE theoretical framework and the csQCA method to analyze 20 CEPI cases in China, leading to the following conclusions.

First, local formalism is influenced by technological, organizational, and environmental factors, including environmental facility constraints, digital facility constraints, accountability pressure, attention constraints, economic constraints, and industrial constraints.

Second, no single condition is necessary for local governments to generate formalism in environmental rectification. The formalism observed in local environmental rectification results from the interplay of multiple conditions. Technological, organizational, and environmental conditions operate in a “multiple and concurrent” manner, forming diverse configurations that drive local governments to adopt formalism. These configurations exhibit the characteristic of “reaching the same destination through different paths”.

Third, three action paths categorized by nine conditional configurations, namely the technology-environment path, technology-organization path, and organization path, are the main driving mechanisms for local governments to generate formalism. The technology-environment path refers to local governments severely constrained by environmental infrastructure and industrial structure, leading to formalism. The technology-organization path, which refers to the constraints of environmental infrastructure and the high pressure of environmental accountability, coupled with the lack of diversified supervision in digital infrastructure construction, induces local governments to generate formalism. The technology-organization path involves constraints in environmental infrastructure, high environmental accountability pressure, and insufficient diversified supervision in digital infrastructure construction, which collectively induce formalism. The organization path includes attention constraints and blame-avoidance thinking. The former refers to local formalism under limited environmental attention, influenced by auxiliary factors such as inadequate infrastructure. The latter arises when local governments, facing environmental protection accountability pressure, develop a blame-avoidance mindset. Driven by this thinking, they are influenced by edge conditions such as industrial constraints and opt for formalism.

Finally, environmental infrastructure constraints, attention constraints, accountability pressure, and industrial constraints serve as core conditions across all pathways, with accountability pressure playing a particularly significant role.

### 5.2 Countermeasures and suggestions

Correcting the formalism of local environmental rectification should be planned comprehensively and targeted. Specific improvement measures can be determined from the perspectives of technology, organization, and environment.

First, improving infrastructure and reducing formalism caused by technical limitations are key measures at the technical level. On the one hand, through increasing financial investments in equipment procurement and maintenance, environmental infrastructure directly related to pollution control can be enhanced. On the other hand, public oversight of local government behavior should be strengthened by expanding digital infrastructure coverage.

Second, at the environmental level, reducing the proportion of the secondary industry, adjusting the industrial layout rationally, and optimizing and upgrading the industrial structure are methods to alleviate environmental rectification pressure and address formalism. During this process, the government should actively implement policies to promote industrial restructuring. Simultaneously, it should emphasize technological innovation and green innovation, leveraging new technologies to transform and upgrade traditional industries, and actively promote high-end, intelligent, and green development in industries.

Third, at the organizational level, preventing lapses in attention and ensuring precise accountability are essential for addressing local formalism. Economic development and environmental protection are equally important. This concept should be established and implemented by local governments. And this can also be set as specific assessment criteria to encourage local governments to focus on environmental protection. Additionally, to alleviate the pressure of accountability, precise accountability mechanisms must be implemented. In individual cases, attention should be paid to the investigation of the facts of the case, and there should be a clear legal basis for whether accountability should be held, who should be held accountable, and to what extent. Accurate accountability also requires strict adherence to fixed accountability procedures. This will prevent confusion and errors caused by program issues. Alongside precise accountability, constraints and incentives should be implemented concurrently to ensure local governments fulfill their responsibilities effectively.

### 5.3 Limitations and prospects

Centering on formalism in local government environmental rectification, this paper employs the TOE framework combined with the csQCA method to identify formative pathways of governmental formalism. This enriches research on local government formalism in CEPI while offering practice solutions. However, local formalism may also be influenced by other external and internal factors, such as the external environment of the rule of law and the environmental values of internal government officials. The inadequacy of this study is that it cannot analyze all the antecedents, but it also provides ideas for further research on the formalism of local governments.
